# Supplementary material for: Collaborative intelligence in AI: Evaluating the performance of a council of AIs on the USMLE
Source: PLOS Digit Health. 2025 Oct 9;4(10):e0000787. doi: 10.1371/journal.pdig.0000787 (PMC12510544; doi:10.1371/journal.pdig.0000787)
Supplement: S2 File — (ZIP) [file pdig.0000787.s002.zip › S2 Data Raw data files containing unprocessed question inputs and LLM outputs including transcripts of deliberations/STEP3_Question-104-1_Type-MC-NJ.docx]

**Facilitator** **[Round 1]:** A 27-year-old nulligravid woman comes to the office because of a 1-month history of daily vaginal bleeding associated with lower abdominal pain and, more recently, shortness of breath. Three days ago, she also developed aching pain of her sternum, which she rates as a 10 on a 10-point scale. During the 6 months prior to her current symptoms, the patient's menstrual periods occurred regularly but were heavy in flow. Her current bleeding has required changing her sanitary pad every 2 hours. Medical history is significant for sickle cell anemia. Medications include hydroxyurea (500 mg daily). The patient appears younger than her stated age. She is 162 cm (5 ft 4 in) tall and weighs 54 kg (120 lb); BMI is 21 kg/m². Vital signs are temperature 37.7°C (99.8°F), pulse 99/min, respirations 19/min, and blood pressure 129/79 mm Hg. Auscultation of the chest discloses coarse breath sounds in both lung bases and a grade 2/6 systolic murmur heard best at the second left intercostal space. Results of laboratory studies are shown: Blood Hematocrit - 18.0% Hemoglobin - 5.7 g/dL RBC - 3.8 million/mm³ WBC - 3000/mm³ Reticulocyte count - <0.4% of red cells Which of the following is the most appropriate management of this patient's sternal pain? (A) Exchange transfusion (B) Increased dose of hydroxyurea (C) Initiation of erythropoietin therapy (D) Iron supplementation (E) Transfusion of packed red blood cells

**AI_A:** RESTATING THE QUESTION: The question describes a 27-year-old woman who exhibits symptoms of heavy vaginal bleeding, abdominal pain, shortness of breath, and severe sternal pain. The patient's medical history reveals that she has sickle cell anemia and has been taking hydroxyurea. Her laboratory results show a severe decrease in hematocrit, hemoglobin, and reticulocyte count, suggesting significant anemia. The task at hand is to determine the most suitable approach to managing this patient's sternum pain. PLANNING: Based on the details provided, it can be inferred that the patient is likely experiencing a vaso-occlusive crisis, a common complication of sickle cell disease. This would be supported by the patient's sternum pain, which could indicate bone marrow infarction. Additionally, the patient's significant vaginal bleeding could cause or exacerbate underlying chronic anemia from her sickle disease, contributing to the symptoms of shortness of breath and possibly heart murmur due to increased cardiac output. Therefore, the most appropriate management for her situation needs to address both the anemic state and the pain crisis. CLINICAL REASONING: From a therapeutic point of view, solving her anemia would not only alleviate her symptoms of shortness of breath and fatigue but also decrease the incidence of bone marrow infarction due to the decreased oxygen delivery in sickle cell disease. This suggests that improving her anemia could also potentially alleviate the sternum pain. Given her lab values, transfusion therapy appears to be a reasonable assumption. CONCLUDING: Based on the given assumptions and clinical correlation, a logical suggestion for managing this patient's sternum pain would be transfusion therapy. This would help in managing the patient's anemia and possibly reduce the sickle cell crisis her body is experiencing. RESPONSE: The most appropriate management of this patient's sternum pain would be (E) Transfusion of packed red blood cells.

**AI_D:** RESTATING THE QUESTION: The case revolves around a 27-year-old woman with a history of sickle cell anemia who is presenting with heavy daily vaginal bleeding leading to severe anemia, manifested by shortness of breath and exertional chest pain. The question is asking what is the best approach to managing her sternal pain. PLANNING: The patient's symptoms might be evidence of several medical disruptions, including a decreased oxygen-carrying capacity due to anemia (hematocrit 18%, hemoglobin 5.7 g/dL), sickle cell vaso-occlusive crises, secondary to hypoxia resultant from anemia, or associated with a potential underlying coagulopathy causing her prolonged bleeding. The lack of reticulocytosis suggests bone marrow inadequacy, possibly due to iron deficiency from chronic blood loss. The options provided allude to different interventions aimed at addressing her sickle cell anemia and possible contributory conditions. CLINICAL REASONING: Exchange Transfusion would be applicable in cases of severe vaso-occlusive crises or acute chest syndrome, which doesn't seem to be the acute issue for this patient at this time. Increasing the dose of Hydroxyurea can boost synthesis of fetal hemoglobin, potentially reducing sickle crises, but this can take time for effectiveness. Erythropoietin stimulates RBC production but is inappropriate here due to the risk of induced polycythemia and thus increasing sickling crises. Iron supplementation could address a potential iron deficiency, but this patient's hematocrit and hemoglobin level, coupled with her physical symptoms, suggest a immediate action is required. CONCLUDING: Given her profound anemia causing clinical symptoms, and concerns for ongoing blood loss, this is a situation requiring swift intervention. Improving her red blood cell count and thus oxygen-carrying capacity will likely help alleviate her symptoms, including the chest pain. RESPONSE: Therefore, the most appropriate intervention would be (E) Transfusion of packed red blood cells to immediately increase her oxygen capacity and alleviate her symptomatic anemia while other investigations and treatments proceed.

**AI_B:** (1) RESTATING THE QUESTION: To rephrase, a nulligravid 27-year-old woman with a history of sickle cell anemia is reporting continuous vaginal bleeding for a month, lower abdominal pain, recent dyspnea, and severe sternal pain that started three days ago. Although she used to have regular but heavy menstrual flow, her current bleeding necessitates changing her sanitary pad every 2 hours. She’s been on hydroxyurea (500 mg daily), but her appearance is younger than her age, and she’s of normal weight and height. She also showed a fever, mild tachycardia, normal respiratory rate, and mild prehypertension, as well as coarse breath sounds in both lung bases and a grade 2/6 systolic murmur. Her lab results point to anemia, leukopenia, and low reticulocyte count, and the challenge lies in determining how her sternal pain can best be managed. (2) PLANNING: Here, the primary assumption is that her sternal pain is due to a vaso-occlusive crisis in sickle cell disease, which can lead to acute chest syndrome or a form of avascular necrosis of the sternum. Meanwhile, secondary assumptions are that her other symptoms may be because of gynecological issues, the side effects of her medication, or her disease itself. But among these assumptions, the most pressing one to address clinically is the first because severe pain on the sternum scored by the patient to be a 10 out of 10 could be an emergency condition related to her primary disease, sickle cell anemia. (3) CLINICAL REASONING: Sickle cell disease is a hemoglobinopathy typified by sickling events where hemoglobin becomes misshapen due to hypoxia, acidosis, or dehydration, among other conditions. This sickling can lead to vaso-occlusion especially in microcirculation. Critical organs such as the lungs, bones including the sternum, and spleen can undergo vaso-occlusive crises, causing painful episodes and acute complications like acute chest syndrome, avascular necrosis, or splenic sequestration. Her lab results show remarkably low hematocrit and hemoglobin, with evidence of insufficient reticulocyte count to compensate for the anemic condition, indicating that a blood transfusion is necessary. (4) CONCLUDING: Chronic anemia combined with heavy menstrual bleeding could have aggravated her inherent state of chronic hemolysis due to sickle cell disease. This would not only heighten her risk of developing sickling crises but also leave her with insufficient oxygen-carrying capacity. Resulting from her increased demand for oxygen, an acute vaso-occlusive crisis could very well explain her severe sternal pain. (5) RESPONSE: Looking at her condition, the most appropriate method to manage her severe pain is transfusion of packed red blood cells, which would not only improve her oxygen-carrying capacity temporarily but also aid in stopping her vaso-occlusive sickling crisis. So the answer is (E) Transfusion of packed red blood cells.

**AI_E:** RESTATING THE QUESTION: The query pertains to a 27-year-old female patient with no prior pregnancies who has been experiencing profuse vaginal bleeding, abdominal and sternum pain, and breathlessness. Over the last half a year, her menstrual cycles have been regular but strong. For the past month, she's had to change her sanitary pad every two hours due to the intensity of the bleeding. Of note, the patient has a history of sickle cell anemia and has been on daily medication with hydroxyurea. She presents with a young appearance, vital parameters within normal limits except slightly raised temperature, and a grade 2/6 systolic murmur heard at the second left intercostal space on chest auscultation. Furthermore, laboratory test results reveal low hematocrit, hemoglobin, WBC, and reticulocyte count levels. PLANNING: The assumptions to consider are as follows: (1) the unchecked vaginal bleeding could have led to her anemia; (2) the sternum pain she is experiencing might be representative of a sickle cell crisis; and (3) the current course of treatment with hydroxyurea is not sufficiently managing her sickle cell anemia. We can question each assumption, thus generating alternative ones. For instance, could the vaginal bleeding be due to a gynecological issue, and not just a manifestation of her overall health decline? Is the sternum pain a symptom of a more serious condition such as myocardial infarction, or pneumonia given the coarse breath sounds noted in the lungs? Is the hydroxyurea dose appropriate, and does it need to be adjusted or supplemented with another form of treatment? Evaluating these, it seems clinically prudent to prioritize the management of her pain and severe anemia, possibly due to a severe sickle cell crisis triggered by her heavy menstrual bleeding. CLINICAL REASONING: Sickle cell anemia is a long-standing, chronic condition manifesting with episodes of intense pain known as sickle cell crises. These crises are precipitated by conditions such as infection, dehydration, stress, and in this patient's case, possibly severe blood loss from intense menstrual bleeding, which can exacerbate anemia. As for the sternum pain, considering the patient's history and the nature of the pain (rated as 10 out of 10), this severe bone pain could be due to vaso-occlusion in the bone marrow, a characteristic of a sickle cell crisis. In such a crisis, the abnormal sickle-shaped red blood cells obstruct blood flow in the small vessels leading to ischemia and subsequent acute chest syndrome and bone pain. The blood work further supports this assumption with low levels of hematocrit (18.0% — indicating severe anemia) and hemoglobin (5.7 g/dL— confirming the severe anemia). Given the severity of these findings, there is a requirement for immediate intervention to alleviate symptoms and restore normal physiology. CONCLUDING: Upon correlating her clinical presentation (profuse bleeding, severe pain, breathlessness) and laboratory findings (severe anemia, low WBC count, inadequate reticulocyte response), we can conclude that the patient's sickle cell disease is not well controlled and that she is likely undergoing a sickle cell crisis aggravated by her heavy menstrual bleeding. The associated severe anemia is likely contributing to her shortness of breath and could be worsening her crisis state. Consequently, the priority is to manage her pain and quickly correct the anemia. RESPONSE: The most suitable management for this patient's sternal pain involves restoring her hemoglobin level to help put an end to the sickle cell crisis. This can be achieved by administering transfusion of packed red blood cells (Option E). The transfusion will increase her hemoglobin level, improving her symptoms and in this case, will also serve to manage her severe bone (sternal) pain.

**AI_C:** This scenario describes a 27-year-old woman with no history of pregnancy who presents with a one-month history of heavy menstrual bleeding, which is known as menorrhagia, along with lower abdominal pain. Her most recent symptom includes sternum pain, described as severe. The patient's periods had been regular but heavy for some months prior to her current symptoms. Related to her hematological health, she is known to have sickle cell anemia and is currently taking hydroxyurea as a therapeutic agent. Despite her age, she appears younger and her BMI is within a healthy range. Vital signs reveal slight fever, a marginally high pulse, and normal respiratory rate and blood pressure. Coarse breath sounds from both lungs and a grade 2/6 systolic murmur suggest possible cardiovascular involvement. Laboratory tests indicate low hematocrit, hemoglobin, RBC count, and WBC count, and an extremely low reticulocyte count. To decide how we should best manage our patient's sternal pain, we need to understand the cause. Some potential causes could include the heavy daily vaginal bleeding leading to severe anemia, sickle cell-induced bone infarcts causing daily sternal pain, or the chronic sickle cell disease leading to a state of chronic pain. We will rule out the option of an increased dose of hydroxyurea because, in clinical practice, dosage adjustments should typically only be made if the patient is not responding to the current dose and given her low WBC count, it would not be appropriate to increase the dosage. Other assumptions that could be challenged include whether the pain is linked to her daily vaginal bleedings or if it's a direct result of the sickle cell disease, or if her chronic pain is due to the underproduction of RBCs causing insufficient oxygen supply to the tissues. Given her severe anemia (hematocrit 18%, hemoglobin 5.7 g/dL, both significantly below the standard range) which can cause her chronic fatigue and shortness of breath, the crucial assumption we should prioritize approaching would likely be the tissues' low oxygenation levels due to underproduction of red blood cells (RBC). Let's review the evidence. First, the woman's restrictive condition (low hematocrit), indicating reduced RBCs volume, and thus, decreased oxygen-carrying capacity. Second, we see her poor condition also reflected in the low WBC count, implying a generally reduced immune response. Given her hydroxyurea medications, myelosuppression (resulting in reduced RBC and WBC count) can occur. However, hydroxyurea is generally not associated with direct sternum pain. Consequently, I would argue that her tissue hypoxia due to severe anemia, which is directly linked to her heavy menstrual bleedings, may be the reason behind her clinical manifestations, particularly her severe sternum pain. She may be experiencing a 'hemolytic crisis' where the rapid destruction of RBCs leads to clinical symptoms such as fatigue, shortness of breath, and chest pain, or severe bone pain. This bone pain could be due to bone infarcts from sickling of cells under hypoxic conditions. The management thus should aim to alleviate this tissue hypoxia by enhancing the oxygen-carrying capacity of the blood, which could potentially reduce her severe sternal pain and other associated symptoms. This can be achieved by swiftly increasing the RBC count and thus the oxygen-carrying capacity of her blood. Therefore, the ideal management of this patient's sternal pain would be option (E), a transfusion of packed red blood cells. This would not only address this patient's sternal pain but also her symptoms of shortness of breath and low energy levels.

**Facilitator** The consensus among the AI responses is that the 27-year-old woman, who has a history of sickle cell anemia and is currently experiencing heavy vaginal bleeding, severe sternal pain, and shortness of breath, is likely undergoing a vaso-occlusive crisis, a common complication of sickle cell disease. This crisis could be exacerbated by her significant vaginal bleeding, which may be causing or worsening her underlying chronic anemia. The severe anemia, indicated by her low hematocrit, hemoglobin, and reticulocyte count, could be contributing to her symptoms of shortness of breath and possibly heart murmur due to increased cardiac output. The sternum pain could be a result of bone marrow infarction due to the vaso-occlusive crisis. Therefore, the management of her situation needs to address both the anemic state and the pain crisis. Given her lab values, transfusion therapy appears to be a reasonable approach. This would not only alleviate her symptoms of shortness of breath and fatigue but also decrease the incidence of bone marrow infarction due to the decreased oxygen delivery in sickle cell disease. Therefore, the most appropriate management of this patient's sternum pain would be transfusion of packed red blood cells (Option E). This would help in managing the patient's anemia and possibly reduce the sickle cell crisis her body is experiencing.
